# Association of systemic immune-inflammation index with malnutrition among Chinese hospitalized patients: a nationwide, multicenter, cross-sectional study

**DOI:** 10.3389/fnut.2024.1375053

**Published:** 2024-08-27

**Authors:** Mengyuan Chen, Shu-an Wang, Jiayao Yang, Jiawang Bai, Jingyue Gu, Haolong Luo, Xudong Zhang, Yan Han, Jihong Shao, Yan Xu, Shuyan Guo, Xiangmei Ren

**Affiliations:** ^1^Department of Nutrition, School of Public Health, Xuzhou Medical University, Xuzhou, Jiangsu, China; ^2^Jiangsu Engineering Research Center of Biological Data Mining and Healthcare Transformation, Xuzhou Medical University, Xuzhou, Jiangsu, China; ^3^Department of Clinic Nutrition, Nanjing Drum Tower Hospital, The Affiliated Drum Tower Hospital Clinical College of Xuzhou Medical University, Nanjing, Jiangsu, China; ^4^Department of Clinic Nutrition, The Affiliated Hospital of Xuzhou Medical University, Xuzhou, China; ^5^National Institute of Hospital Administration, National Health Commission, Beijing, China; ^6^Jiangsu Provincial Center for Disease Control and Prevention, Nanjing, Jiangsu, China

**Keywords:** systemic immune-inflammation index, malnutrition, NRS 2002, GLIM, CONUT score

## Abstract

**Background:**

Systemic immune-inflammation index (SII) is associated with increased risk in a wide range of illnesses. However, few studies have explored the associations between SII and the risk of malnutrition. Therefore, this study aimed to investigate the association between SII and malnutrition in a nationwide, multicenter, cross-sectional study involving Chinese hospitalized patients.

**Design:**

From August 2020 to August 2021, a total of 40,379 hospitalized patients met the inclusion and exclusion criteria. Detailed demographic data, diagnoses, as well as physical and laboratory examination results were recorded. The diagnosis of malnutrition was used with two distinct methods: the Malnutrition Screening Tool 2002 (NRS 2002) + Global Leaders Initiative on Malnutrition (GLIM) criteria and the controlling nutritional status (CONUT) score. The risk factors for malnutrition were analyzed using binary logistic regression and multiple logistic regression to obtain odds ratios (OR) and 95% confidence intervals (CI). Restricted cubic spline (RCS), linear spline, and receiver operating characteristic (ROC) analysis were also used.

**Results:**

The prevalence of malnutrition diagnosed by the two methods was 13.4% and 14.9%, respectively. In the NRS 2002 + GLIM diagnostic model, lnSII showed statistical significance between the malnutrition and non-malnutrition group (6.28 ± 0.78 vs. 6.63 ± 0.97, *p* < 0.001). A positive association was observed between higher SII and the risk of malnutrition in both before and after adjustment models compared to the first quartile (Q_3_ vs. Q_1_, OR = 1.27, 95%CI: 1.15–1.40; Q_4_ vs. Q_1_, OR = 1.83, 95%CI: 1.67–2.00). However, a significant reduction in prevalence was observed when SII was in the second quartile (Q_2_ vs. Q_1_, OR < 1), as indicated by a restricted cubic spline with a U trend (*p* for nonlinear <0.001). According to the CONUT score, the prevalence of individuals with normal nutritional status decreased with increasing SII, while the occurrence of three different degrees of malnutrition generally increased. The Kappa value between the two diagnostic methods was 0.23, and the merged data observed an area under the ROC curve of 0.73 (95%CI: 0.714–0.742).

**Conclusion:**

The U-shaped association between SII and the prevalence of malnutrition was observed. Both lower and higher SII levels (either continuous or categorical variable) were significantly associated with an increased risk of malnutrition.

## 1 Introduction

Malnutrition is a major public health problem, posing a substantial risk for adverse clinical outcomes, which is common among hospitalized patients in China and other countries (1, 2). The prevalence of clinical malnutrition diagnosis varies from 5.0% to 25.5%, with an incidence exceeding 50% in cases of malignant diseases (3–5). Despite variations due to different screening tools and disease types (6), numerous studies have shown that malnutrition is correlated with increased mortality, complications, prolonged hospital stays, and decreased quality of life among hospitalized patients (7, 8).

Inflammation significantly contributes to malnutrition (9). Weight loss is widely recognized as a key clinical manifestation of malnutrition and serves as a fundamental criterion in various screening tools (10). The Global Leadership Initiative on Malnutrition (GLIM) criteria, a method that promises to unify the diagnosis of malnutrition globally, includes inflammation as a key component for diagnosing malnutrition. According to GLIM criteria, inflammation could be identified through chronic inflammation-related disease burden or clinical indicators of acute or severe inflammation (11). Inflammatory processes disrupt normal metabolic pathways, increasing energy expenditure and altering nutrient utilization (12). Elevated pro-inflammatory cytokines, like IL-18 and TNF-α, induce catabolic effects, leading to muscle wasting and weight loss (13). Inflammation also impacts appetite regulation and the gastrointestinal system, reducing food intake and altering nutrient absorption (14, 15). However, it should be noted that these static inflammatory markers are relatively limited in their ability to monitor disease progression and the effectiveness of nutritional interventions over time.

The systemic immune-inflammation index (SII), introduced by Hu et al. (16), is a comprehensive inflammatory biomarker reflecting both localized immune responses and systemic inflammation. Studies have shown associations between SII and disease progression and prognosis in cardiovascular disease, cancer, and other conditions (17, 18). However, there is a lack of consensus in malnutrition studies. A study indicated that the predictive performance of SII for malnutrition in hospitalized COVID-19 patients was unsatisfactory (19), while other research highlighted significant associations between malnutrition and inflammation markers. For instance, the geriatric nutritional risk index (GNRI) correlated strongly with inflammatory indicators (20). Immune nutrition therapy has effectively reduced inflammatory markers and prevented malnutrition (21). SII effectively reflects the immune and inflammatory status in various pathological conditions linked with malnutrition. The integration of immune cell counts in SII offers a dynamic and responsive measure of inflammation compared to static markers like CRP or IL-6 (22). The ability of SII to integrate multiple hematological parameters allows a more comprehensive assessment of the inflammatory state, thereby improving the accuracy of the diagnosis of malnutrition and sarcopenia. Studies have demonstrated significant associations between SII and nutritional status in various patient populations, suggesting SII as a valuable tool for identifying at-risk individuals and tailoring interventions (23).

It is evident that SII offers a more comprehensive and integrative approach to assessing inflammation compared to the GLIM criteria, potentially providing earlier and more sensitive detection of inflammation-induced malnutrition (24). The GLIM criteria offered a structured framework for diagnosing malnutrition using specific inflammatory markers. However, SII, with its holistic and dynamic assessment of immune-inflammatory status, represented a significant advancement in understanding and managing the complex relationship between inflammation and malnutrition. Therefore, the objective of this study is to investigate the relationship between SII and the risk of malnutrition in hospitalized patients using multicenter data and stratified sampling in China, aiming to provide a more scientific theoretical basis for future prevention and intervention measures.

## 2 Methods

### 2.1 Data source

A multistage stratified cluster random sampling was employed to assess the nutritional status of hospitalized patients in China from August 2020 to August 2021.

#### 2.1.1 Multistage sampling

First stage: Peking Union Medical College Hospital was selected as the general lead hospital. Additionally, 29 provincial lead hospitals were designated across 31 provinces (including autonomous regions and municipalities) to coordinate case investigation and data reporting.

Second stage: Each of the 30 lead hospitals (Peking Union Medical College Hospital and the 29 provincial lead hospitals) established 1–23 satellite hospitals within their respective administrative regions, creating a two-tier system of lead hospitals (coordinators) and satellite hospitals (executors).

#### 2.1.2 Stratified sampling

Within each province, autonomous region, and municipality, hospitals were stratified based on their classification, ensuring that only hospitals classified as Class II Grade A or above were selected.

#### 2.1.3 Cluster sampling

Within each selected hospital, newly hospitalized patients who met the inclusion and exclusion criteria were consecutively selected for investigation until the predetermined total of 200 cases per hospital was reached. Basic nutritional data of these newly hospitalized patients with seven specialized system diseases (tumor, endocrine diseases, nervous diseases, circulatory diseases, respiratory diseases, digestive diseases, and urogenital diseases) were collected.

This method ensured a comprehensive and representative sample of hospitalized patients across different regions and hospital grades in China. The study underwent a thorough review and received approval from the Ethics Committee of the Peking Union Medical College Hospital (No. ZS-2614). All patients provided informed consent by signing appropriate documentation before participating in the survey.

### 2.2 Sample size estimation

The sample size was determined based on the overall incidence of nutritional risk among 6,638 hospitalized patients in 34 hospitals in China in 2017, which was 42.34% (25). The sample size for each stratum was calculated using the following formula.


$$N=\frac{Z^{2}\;\left[P\left(1-P\right)\right]}{E^{2}}$$

The acceptable error range, denoted E, was established at ±1% (*E* = 0.01). The standard deviation corresponding to a 95% confidence level, denoted *Z*, was approximately rounded to 1.96 (*Z* = 2). The empirical statistic for the total incidence of nutritional risk in hospitalized patients, denoted as *P*, was 0.4234. By substituting these values into the formula, the sample size could be calculated as *N* = 9,765. Taking into account a follow-up loss rate of 20%, we estimated that each stratum would require a total of 11,718 individuals. This survey was stratified based on three regions: eastern, central, and western; therefore, a total of 35,154 patients were required for this study.

### 2.3 Inclusion and exclusion criteria

The inclusion criteria were as follows: (1) patients newly admitted to the hospital with diseases in any of the seven specialty systems; (2) age ≥ 18 years old; and (3) within 24–48 h after admission.

The exclusion criteria encompassed the following: (1) pediatric and critically ill patients; (2) individuals with mental illness or memory disorders that impeded accurate response to questions; (3) those lacking behavioral capacity; and (4) other conditions deemed unsuitable for inclusion by the investigators.

### 2.4 Evaluation of SII

The calculation formula for SII, which is based on peripheral neutrophil count (N), lymphocyte count (L), and platelet count (P), can be mathematically expressed as SII = N × P/L. Additionally, the SII was transformed into the natural logarithm (lnSII) and stratified by the interquartile range (IQR) to meet the data analysis requirements in our study.

### 2.5 Diagnosis of malnutrition

**Figure 1:**
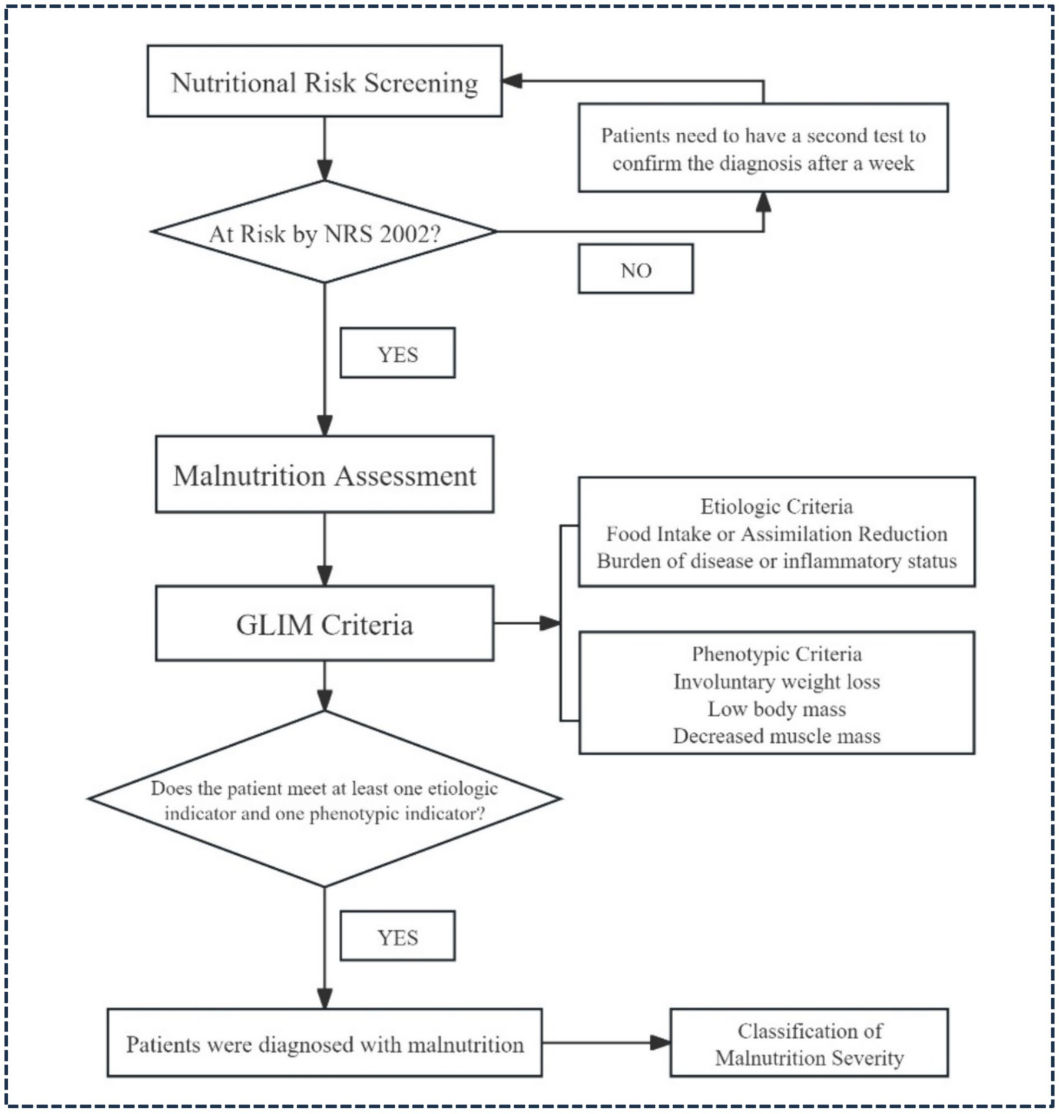
Flow chart of the NRS 2002 + GLIM criteria for the diagnosis of malnutrition.

The diagnosis of malnutrition in this study consisted of two primary steps: nutritional risk screening and malnutrition assessment. Figure 1 presents a diagnostic flow chart that illustrates the identification process. Initially, nutritional risk screening was followed by a comprehensive evaluation using the Malnutrition Screening Tool 2002 (NRS 2002) to identify patients at risk (score ≥ 3) (26). For details on the NRS 2002 assessment and results, please refer to Supplementary Tables S1, S2. Subsequently, an assessment was performed based on the Global Leaders Initiative on Malnutrition (GLIM) criteria, requiring patients to meet both phenotypic and etiologic indicators for a confirmed diagnosis (11). Supplementary Table S3 shows the detailed diagnosis of malnutrition.

To validate the efficacy of SII compared to other nutritional screening tools, we introduced the controlling nutritional status (CONUT) score in this study. The CONUT score is an additional screening tool that can objectively and simply assess the nutritional status of hospitalized patients. It comprises three indicators: serum albumin (ALB), total cholesterol (TC), and peripheral lymphocyte count. Among them, albumin reflects protein reserves; total cholesterol is associated with caloric deficiency, and lymphocyte count is related to immune defense (27). Unlike the NRS 2002 + GLIM criteria diagnostic model, the CONUT score does not take into account anthropometric measures that may be influenced by effusion and edema, such as body mass index (BMI), upper arm circumference, and calf circumference (28). The scoring criteria are determined based on the ranges of the three indicators, and the CONUT score is the sum of the three scores. As presented in Table 1, four groups of patients with varying degrees of undernutrition were defined based on the CONUT score: normal (0–1), mild (2–4), moderate (5–8), and severe (9–12).

**Table 1:** Controlling nutrition status score calculation.

Parameters	Undernutrition degree			
	Normal	Mild	Moderate	Severe
Serum albumin (g/L)	≥35	30–34.9	25–29.9	<25
Score	0	2	4	6
Total lymphocytes (×10^9^/L)	≥1.6	1.2–1.59	0.8–1.19	<0.8
Score	0	1	2	3
Cholesterol (mg/dL)	≥180	140–179	100–139	<100
Score	0	1	2	3
Screening total score	0–1	2–4	5–8	9–12

### 2.6 Definition of covariates

Furthermore, our study incorporated covariates that may influence outcomes. Sociodemographic factors included gender, age, and education levels. Physical examination included BMI, waist circumference, hip circumference, upper arm circumference, calf circumference, and grip strength. Blood test indices comprised white blood cell count (WBC), red blood cell count (RBC), hemoglobin (Hb), total cholesterol (TC), triglyceride (TG), and albumin (ALB). Measurement methods of physical examination indicators and normal reference value range of blood test indices can be found in Supplementary Tables S4, S5.

### 2.7 Statistical analysis

Data imputations and analyses were performed using IBM SPSS Statistics (version 27.0) and RStudio (version 4.3.0), utilizing packages such as “rms” for regression modeling, “ggplot2” for graphic visualization, and “pROC” for ROC curve analysis.

Multiple imputation was employed to address missing values in this study. By comparing the data before and after imputation, the optimal dataset was selected for handling missing values. Categorical variables were described using rates or composition ratios (%), and comparisons between groups for unordered variables were made using the chi-square test. Continuous variables following a normal distribution were described as mean ± standard deviation (SD) and compared using the *t*-test. For non-normally distributed continuous variables, the median (P_25_–P_75_) was used, and comparisons were made using the Mann–Whitney U test. All tests were two-tailed, with a significance level of *α* = 0.05.

A series of stepwise logistic regression models were conducted to explore the relationship between SII and malnutrition. The crude model did not incorporate any covariates. Model I included sociodemographic characteristics. Model II further adjusted for physical examination characteristics, and Model III additionally accounted for disease characteristics.

Restricted cubic spline (RCS) was employed to visually represent the dose–response relationship between SII and malnutrition risk, elucidating potential nonlinearity. To avoid overfitting with RCS, linear spline was also used to simulate their linear association. This comprehensive approach facilitated an accurate assessment of the link between SII and malnutrition.

To assess the suitability of SII in the CONUT score, multiple logistic regression was used to explore potential risk factors associated with varying degrees of malnutrition. SII was categorized into quartiles, with the first quartile (Q_1_) as the reference. Normal nutritional status served as the reference for the dependent variable. Models were iteratively adjusted, odds ratios (ORs) were calculated, and corresponding forest plots were generated.

In the consistency analysis, we validated the effectiveness of two methods for diagnosing malnutrition by calculating the Kappa value and analyzing consistency data. A Kappa value of 0.21–0.40 indicated fair agreement, 0.41–0.60 moderate agreement, and 0.61–0.80 substantial agreement. The predictive ability of SII within the consistency data was compared using receiver operating characteristic (ROC) analysis, and the area under the curve (AUC) was calculated.

## 3 Results

### 3.1 Baseline characteristics according to the NRS 2002 + GLIM criteria for the diagnosis of malnutrition

The baseline characteristics of the subjects are provided in Table 2. A total of 40,379 participants were included in this study. Among them, 56.7% were men, with a mean age of 59.6 ± 14.3 years, and more than half had primary or middle school education. Circulatory diseases had the highest prevalence among the seven system diseases, accounting for 36.1%, while urogenital diseases had the lowest prevalence at 15.2%. The diagnosis of malnutrition was established using the NRS 2002 + GLIM criteria, identifying a total of 5,400 patients with malnutrition, resulting in a prevalence of approximately 13.4%. Individuals with malnutrition exhibited a higher likelihood of being male, having less than a junior high school education, and comorbidities such as tumors, respiratory diseases, and digestive diseases. Additionally, malnourished patients showed higher levels of SII, age, and WBC; while demonstrating lower BMI, waist circumference, hip circumference, upper arm circumference, calf circumference, and grip strength; as well as reduced levels of RBC and Hb along with decreased TC, TG, and ALB.

**Table 2:** Baseline characteristics of the study population stratified by malnutrition.

Characteristics	Total subjects	Malnutrition		*p*-value
		No	Yes	
Participants, *n (%)*	40,379 (100.0)	34,979 (86.6)	5,400 (13.4)	
lnSII	6.33 ± 0.81	6.28 ± 0.78	6.63 ± 0.97	<0.001
**SII,** *n (%)*				<0.001
Q_1_ (<332)	10,085 (25.0)	9,098 (90.2)	987 (9.8)	
Q_2_ (332–514)	10,125 (25.1)	9,219 (91.1)	906 (8.9)	
Q_3_ (515–874)	10,079 (24.9)	8,781 (87.1)	1,298 (12.9)	
Q_4_ (>875)	10,090 (25.0)	7,781 (78.1)	2,209 (21.9)	
**Gender,** *n (%)*				<0.001
Male	22,894 (56.7)	19,680 (86.0)	3,214 (14.0)	
Female	17,485 (43.3)	15,299 (87.5)	2,186 (12.5)	
Age, *years*	59.6 ± 14.3	58.7 ± 14.1	65.3 ± 15.0	<0.001
**Education Level,** *n (%)*				<0.001
Never	4,303 (10.7)	3,466 (80.5)	837 (19.5)	
Primary or junior high school	20,523 (50.8)	17,668 (86.1)	2,855 (13.9)	
High school or vocational school	9,930 (24.6)	8,738 (88.0)	1,118 (12.0)	
Bachelor’s degree or above	5,623 (13.9)	5,107 (90.8)	495 (9.2)	
**Physical indicators**				
BMI, kg/m^2^	23.9 ± 3.9	24.3 ± 3.7	20.7 ± 3.8	<0.001
Waist circumference, cm	86.9 (79.7, 94.0)	88.0 (80.5, 95.0)	79.5 (71.3, 87.0)	<0.001
Hip circumference, cm	94.8 (89.3,100.0)	95.2 (90.0, 101.0)	88.9 (82.9, 94.5)	<0.001
Biceps circumference, cm	27.8 ± 3.9	28.2 ± 3.7	25.0 ± 4.0	<0.001
Calf circumference, cm	33.8 ± 4.6	34.3 ± 4.4	30.7 ± 4.6	<0.001
Grip strength, kg	25.3 (18.6, 33.2)	26.1 (19.3, 34.0)	20.5 (14.6, 27.8)	<0.001
**Laboratory indicators**				
WBC, ×10^9^/L	6.12 (4.90, 7.77)	6.11 (4.91, 7.69)	6.21 (4.70, 8.45)	<0.001
RBC,×10^12^/L	4.31 (3.83, 4.74)	4.36 (3.92, 4.78)	3.88 (3.35, 4.38)	<0.001
Hb, g/L	131 (116, 145)	132 (119, 145)	117 (100, 132)	<0.001
TC, mmol/L	4.32 (3.57, 5.14)	4.37 (3.62, 5.17)	4.01 (3.30, 4.84)	0.496
TG, mmol/L	1.27 (0.90, 1.86)	1.30 (0.92, 1.91)	1.10 (0.81, 1.55)	<0.001
ALB, g/L	39.8 (36.3, 43.0)	40.2 (37.0, 43.4)	36.2 (32.0,40.0)	<0.001
**Tumor,** *n (%)*				<0.001
No	31,529 (78.1)	28,156 (89.3)	3,373 (10.7)	
Yes	8,850 (21.9)	6,823 (77.1)	2,027 (22.9)	
**Endocrine disease,** *n (%)*				<0.001
No	29,974 (74.2)	25,609 (85.4)	4,365 (14.6)	
Yes	10,405 (25.8)	9,370 (90.1)	1,035 (9.9)	
**Nervous disease,** *n (%)*				<0.001
No	33,609 (83.2)	28,964 (86.2)	4,645 (13.8)	
Yes	6,770 (16.8)	6,015 (88.8)	755 (11.2)	
**Circulation disease,** *n (%)*				<0.001
No	25,802 (63.9)	22,068 (85.5)	3,734 (14.5)	
Yes	14,577 (36.1)	12,911 (88.6)	1,666 (11.4)	
**Respiratory disease,** *n (%)*				<0.001
No	33,319 (82.5)	29,400 (88.2)	3,919 (11.8)	
Yes	7,060 (17.5)	5,579 (79.0)	1,481 (21.0)	
**Digestive disease,** *n (%)*				<0.001
No	31,881 (79.0)	27,996 (87.8)	3,885 (12.2)	
Yes	8,498 (21.0)	6,983 (82.2)	1,515 (17.8)	
**Genitourinary disease,** *n (%)*				<0.001
No	34,254 (84.8)	29,503 (86.1)	4,751 (13.9)	
Yes	6,125 (15.2)	5,476 (89.4)	649 (10.6)	

Data were presented as *n* (%), mean ± SD or median (P_25_–P_75_); SII, Systemic immune-inflammatory index; BMI, Body mass index; WBC, White blood cell count; RBC, Red blood cell count; Hb, Hemoglobin; TC, Total cholesterol; TG, Triglyceride; ALB, Albumin.

Furthermore, Table 2 presents the results of the *t*-tests and the chi-square tests conducted for each variable. All covariates, with the exception of TC, exhibited a significant association with malnutrition (*p* < 0.05). Among the variables under investigation, lnSII was found to be significantly higher in the malnutrition group compared to the non-malnutrition group (6.28 ± 0.78 vs. 6.63 ± 0.97, *p* < 0.001). Furthermore, a clear trend emerged in the prevalence of malnutrition when converting SII to IQR. Initially, there was a downward trend, but this was followed by an increase as SII rose from Q_2_ to Q_4_. Notably, the lowest prevalence was observed at 8.9% when SII was in Q_2_.

### 3.2 Logistic regression models to assess the association between SII and the risk of malnutrition

To further validate the association between SII and the risk of malnutrition, a stepwise binary logistic regression model was constructed, as delineated in Table 3. In the crude model, participants in Q_2_ (OR = 0.91, 95%CI: 0.82–0.99) exhibited a significantly lower risk of malnutrition compared to those in Q_1_. However, with the elevation of SII to Q_3_ and Q_4_, there was a substantial increase in risk with corresponding ORs of 1.36 (95%CI: 1.25–1.49) and 2.58 (95%CI: 2.39–2.80), respectively. The results from Model I and Model II remained robust and statistically significant even after adjusting for multiple variables through multivariate analysis. However, with the introduction of disease characteristic variables in Model III, the statistical significance in Q_2_ was no longer evident (OR = 0.94, 95% CI: 0.85–1.05). Nevertheless, the general associations in the four models remained largely unchanged, indicating a correlation between higher levels of SII and an increased risk of malnutrition.

**Table 3:** Relationship between SII and malnutrition among Chinese hospitalized patients.

Characteristics	OR (95%CI)			
	Crude model	Model I	Model II	Model III
**SII category (Ref, Q_1_ < 332)**				
Q_2_ (332–514)	0.91 (0.82, 0.99)	0.89 (0.81, 0.98)	0.89 (0.81, 0.99)	0.94 (0.85, 1.05)^NS^
Q_3_ (515–874)	1.36 (1.25, 1.49)	1.32 (1.21, 1.45)	1.23 (1.11, 1.35)	1.27 (1.15, 1.40)
Q_4_ (>875)	2.58 (2.39, 2.80)	2.42 (2.23, 2.63)	1.82 (1.66, 1.99)	1.83 (1.67, 2.00)
**Gender (Ref, Male)**				
Female		0.88 (0.83, 0.94)	0.75 (0.70, 0.81)	0.78 (0.73, 0.84)
**Age Group (Ref, 18–44)**				
45–64		0.97 (0.87, 1.08) ^NS^	1.08 (0.96, 1.21)^NS^	0.93 (0.83, 1.05)^NS^
65+		2.24 (2.02, 2.49)	2.08 (1.85, 2.34)	1.91 (1.69, 2.16)
**Education level (Ref, Never)**				
Primary or junior high school		0.79 (0.72, 0.86)	0.90 (0.81, 0.99)	0.87 (0.79, 0.96)
High school or vocational school		0.77 (0.70, 0.86)	0.96 (0.86, 1.08)^NS^	0.95 (0.84, 1.07)^NS^
Bachelor’s degree or above		0.64 (0.56, 0.73)	0.87 (0.75, 1.00)^NS^	0.87 (0.75, 1.01)^NS^
**BMI category (Ref, Normal)**				
Underweight			6.26 (5.69, 6.89)	6.06 (5.50, 6.68)
Overweight			0.50 (0.46, 0.55)	0.54 (0.49, 0.59)
Obese			0.42 (0.37, 0.48)	0.47 (0.41, 0.54)
**BC category (Ref, Normal)**				
Below normal range			1.58 (1.46, 1.72)	1.53 (1.41, 1.67)
**CC category (Ref, Normal)**				
Below normal range			1.60 (1.46, 1.75)	1.65 (1.51, 1.81)
**Grip category (Ref, Normal)**				
Below normal range			1.67 (1.55, 1.79)	1.68 (1.56, 1.80)
**Tumor (Ref, No)**				
Yes				2.28 (2.38, 2.79)
**Endocrine disease (Ref, No)**				
Yes				1.24 (1.14, 1.35)
**Nervous disease (Ref, No)**				
Yes				1.10 (1.00, 1.22)^NS^
**Circulation disease (Ref, No)**				
Yes				1.10 (1.00, 1.17)^NS^
**Respiratory disease (Ref, No)**				
Yes				1.46 (1.34, 1.60)
**Digestive disease (Ref, No)**				
Yes				1.83 (1.69, 1.99)
**Genitourinary disease (Ref, No)**				
Yes				1.06 (0.95, 1.18)^NS^

NS, No statistical significance; OR, Odds ratio; 95%CI, 95% Confidence interval; SII, Systemic immune-inflammatory index; BMI, Body mass index; BC, Biceps circumference; CC, Calf circumference. Model I: Adjusted for sociodemographic characteristics. Model II: Adjusted for Model I and physical examination characteristics. Model III: Adjusted for Model II and disease characteristics.

### 3.3 Linear and nonlinear association between SII and the risk of malnutrition

**Figure 2:**
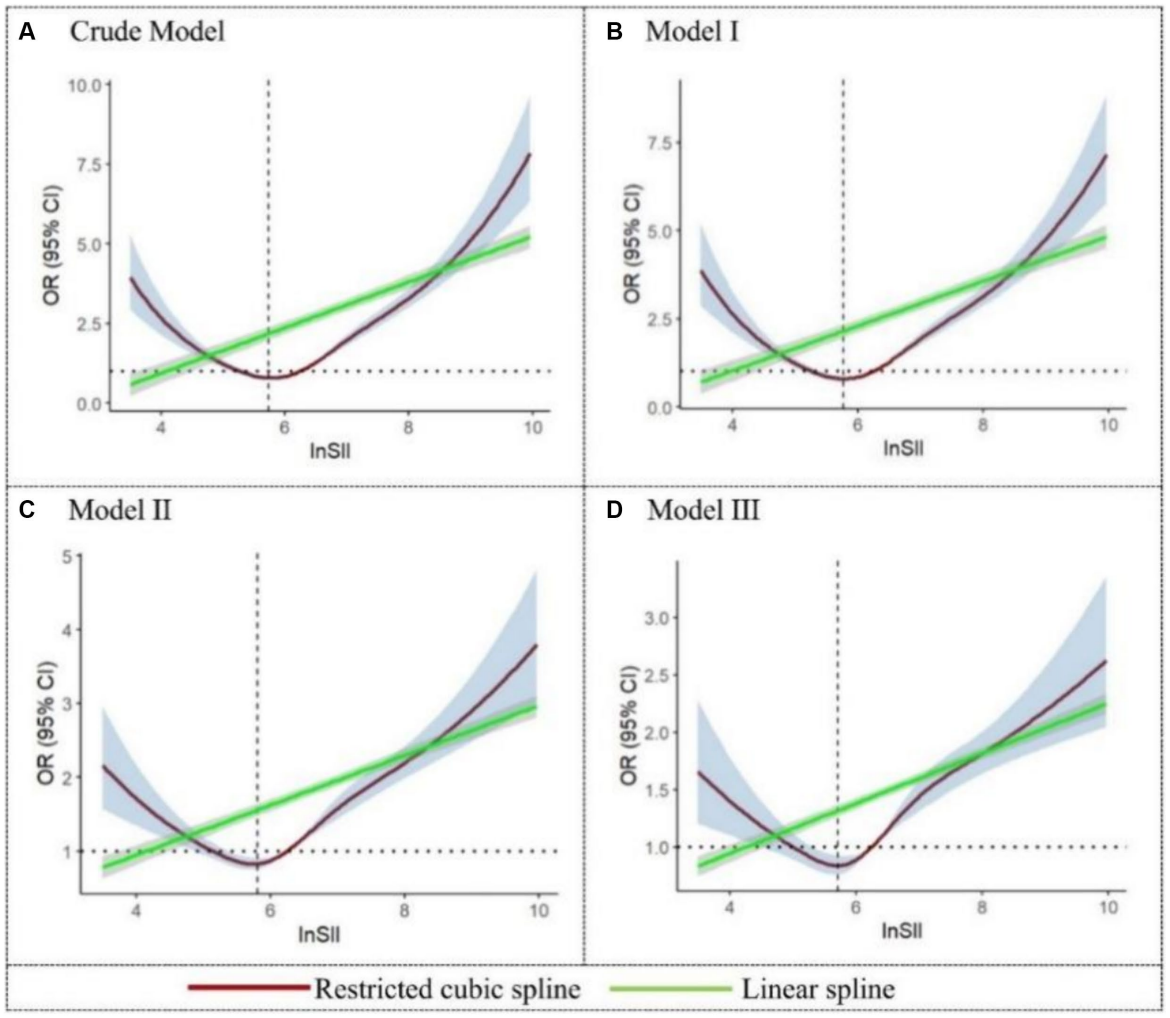
Restricted cubic spline and linear spline for the relationship between SII and the risk of malnutrition. The 95%CI of the adjusted OR in the restricted cubic spline was visually represented by the blue-shaded area, while the gray-shaded area represents the 95%CI of the OR in the linear spline.

As illustrated in Figure 2, all four models demonstrated a significant nonlinear association between SII and malnutrition (P for nonlinear <0.001). In the crude model, the RCS depicted a negative relationship between lnSII and malnutrition until it reached the lowest point at a lnSII level of 5.77 (OR = 0.79, 95%CI = 0.72–0.86); thereafter, the gradual increase in the level of lnSII was associated with an increased risk of malnutrition, revealing a U-shaped dose–response pattern. A similar trend was also evident in the adjusted model, where the OR value reached its minimum around a lnSII of 5.75 (corresponding to SII = 315 × 10^9^ cells/μL). However, linear spline fitting in all four figures consistently indicated a continuous rise in the risk of malnutrition as lnSII increased.

**Figure 3:**
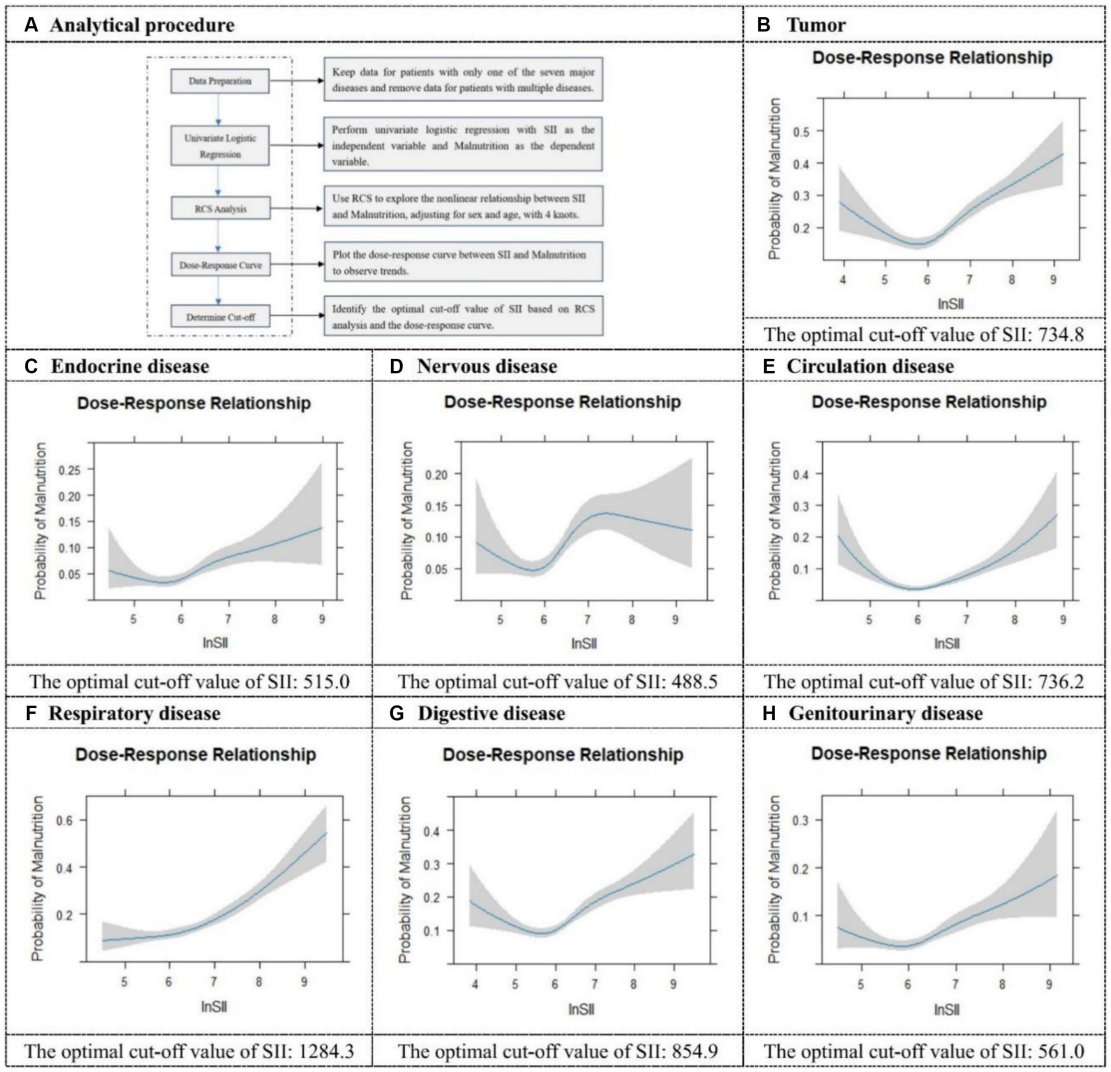
Restricted cubic spline plots and cut-off values for seven different categories of diseases. It illustrates the analytical procedure for determining the optimal cut-off value **(A)**, and provides specific optimal cut-off values for SII in tumor **(B)**, endocrine disease **(C)**, nervous disease **(D)**, circulation disease **(E)**, respiratory disease **(F)**, digestive disease **(G)**, and genitourinary disease **(H)**.

Considering the potential interactions between different types of diseases, we further analyzed the cut-off values of SII in cases where malnutrition was present and only one type of disease was involved. Specifically, for seven different types of diseases, the cut-off values of SII in the presence of malnutrition were presented in Figure 3. Among these, the cut-off value for respiratory diseases (SII = 1284.4 × 10^9^ cells/μL) is the highest, indicating that patients with respiratory diseases have the strongest inflammatory response in the presence of malnutrition. Conversely, the cut-off value for neurological disorders (SII = 488.5 × 10^9^ cells/μL) is the lowest, suggesting that malnourished patients with neurological disorders have a comparatively lower inflammatory response. These results suggested that the cut-off values of SII vary significantly among patients with different diseases when malnutrition is present. Such differences reflected the varying impacts of malnutrition on the inflammatory response in different disease states. The ROC results for the seven diseases were presented in Supplementary Figure S1.

### 3.4 Different severity of malnutrition according to the CONUT score

The descriptive characteristics of the 40,379 participants based on their CONUT scores were presented in Table 4 (The full table was provided in Supplementary Table S6). In general, the prevalence of patients with normal and mild malnutrition was 40.1 and 45.0%, respectively, with the remaining 14.9% displaying moderate and severe malnutrition. It could be observed that among participants without malnutrition, there was a decrease in the prevalence of normal individuals from 51.9% to 17.6% as SII increased from Q_1_ to Q_4_. On the contrary, contrasting results were evident within the malnutrition population, where an increase in SII corresponded to an overall rise in malnutrition prevalence, indicating a positive correlation trend. Interestingly, a U-shaped trend in prevalence was also observed in patients with moderate and severe malnutrition compared to those who were normal or mildly malnourished. Likewise, the prevalence reached its minimum when SII was in Q_2_.

**Table 4:** Baseline characteristics of the study population stratified by the CONUT score (Part).

Characteristics	Undernutrition degree			
	Normal	Mild	Moderate	Severe
Participants, *n (%)*	16,202 (40.1)	18,162 (45.0)	5,286 (13.1)	729 (1.8)
lnSII	6.07 ± 0.59	6.41 ± 0.81	6.77 ± 1.03	6.99 ± 1.22
**SII, *n (%)***				
Q_1_ (<332)	5,236 (51.9)	3,872 (38.4)	864 (8.6)	113 (1.1)
Q_2_ (332–514)	5,133 (50.7)	4,243 (41.9)	673 (6.6)	76 (0.8)
Q_3_ (515–874)	4,056 (40.2)	4,774 (47.4)	1,138 (11.3)	111 (1.1)
Q_4_ (>875)	1,777 (17.6)	5,273 (52.3)	2,611 (25.9)	429 (4.3)

**Figure 4:**
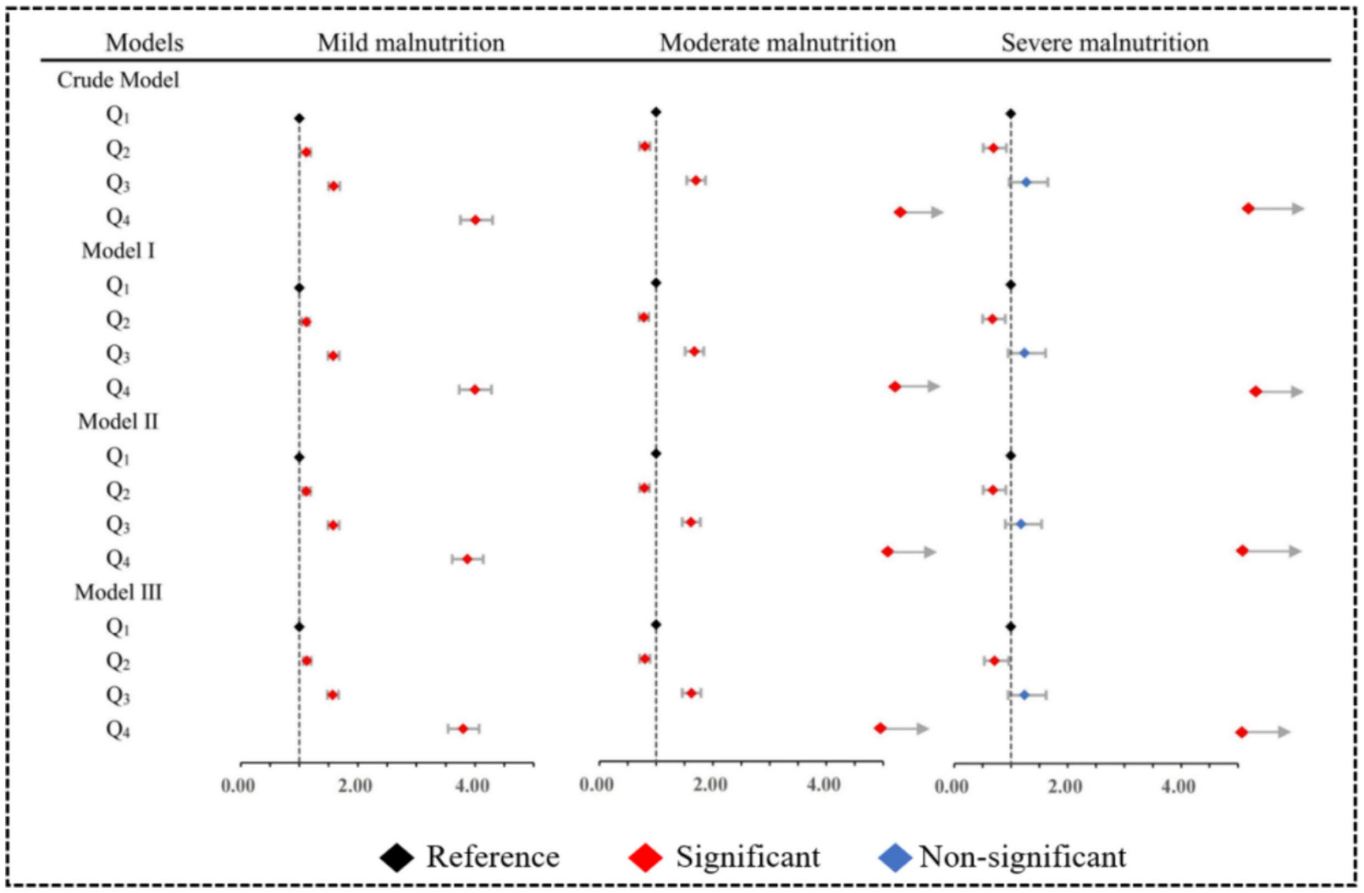
Multiple model adjusted forest plots for different severities of malnutrition. Model I: Adjusted for sociodemographic characteristics. Model II: Adjusted for Model I and physical examination characteristics. Model III: Adjusted for Model II and disease characteristics.

We used forest plots to visualize multiple logistic regression results for different levels of malnutrition (Figure 4). In mild malnutrition, elevated SII was a risk factor in all four models before and after adjustment (OR > 1). Although among moderate malnutrition cases, SII within Q_2_ exhibited a potential protective factor compared to Q_1_ (OR < 1). Similar findings were observed in cases of severe malnutrition. However, the adjusted model revealed that when SII was in Q_3_, it no longer exhibited statistical significance in severe malnutrition, while there was still a tendency for an increased risk of malnutrition observed in Q_4_. The specific OR could be found in Supplementary Table S7.

### 3.5 Consistency test between the NRS 2002 + GLIM criteria and the CONUT score

**Figure 5:**
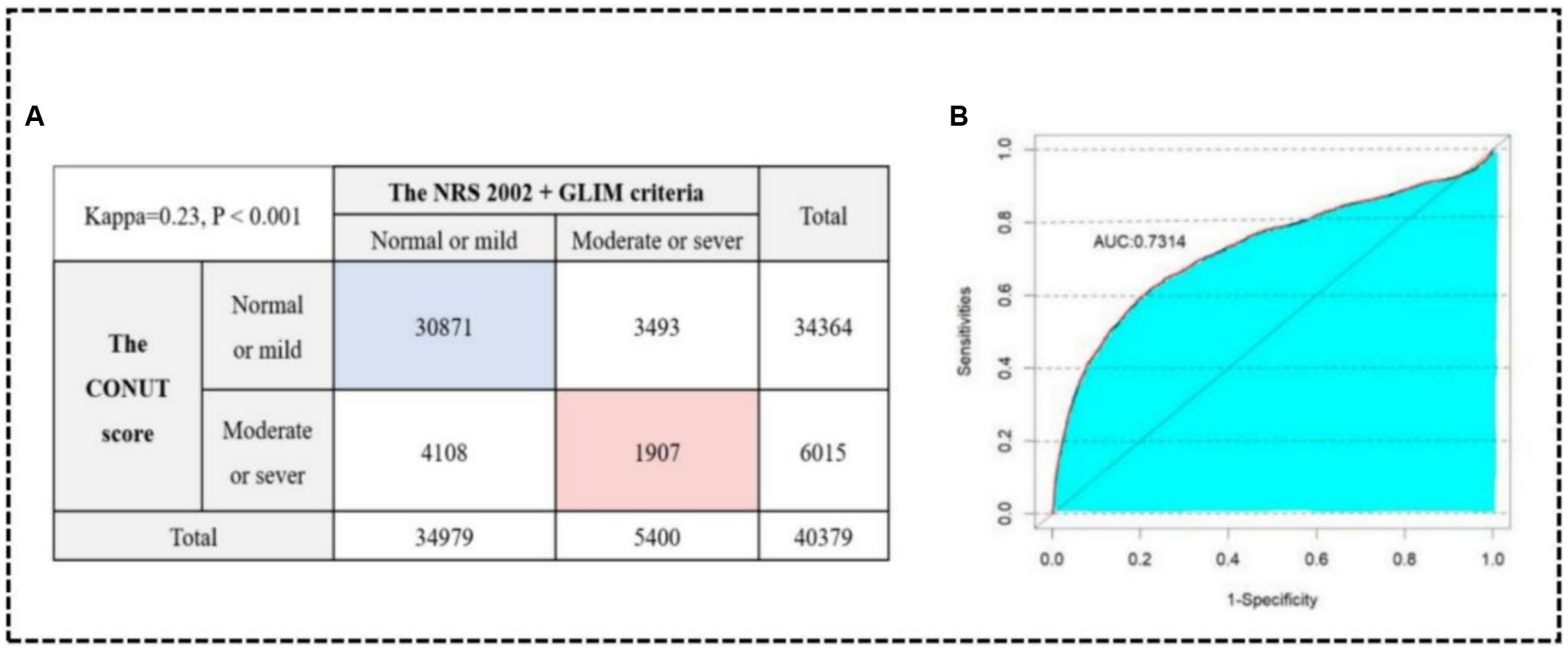
Kappa consistency test between the NRS 2002 + GLIM criteria and the CONUT score **(A)**. ROC curve in the consistency data **(B)**.

To validate the diagnostic effectiveness of the two methods, patients with normal and mild malnutrition on the CONUT score were combined into a group, while those with moderate and severe malnutrition were grouped, ensuring consistency with the grouping basis of the NRS 2002 + GLIM criteria (29). The results of the Kappa consistency test (Kappa = 0.23) revealed a low level of agreement between the severity of malnutrition determined by the NRS 2002 + GLIM criteria and the CONUT score (Figure 5A). However, SII performed well in both methods, indicating its effectiveness in prediction. Finally, the consistency data obtained from both methods were combined and a ROC analysis was conducted on the merged dataset (Figure 5B). The resulting area under the curve was 0.73 (95%CI: 0.714–0.742), indicating the stability of our findings.

## 4 Discussion

In this nationally representative cross-sectional study, the main findings were as follows: (1) Malnutrition was relatively common among hospitalized patients in China, with a prevalence of 13.4 and 14.9% diagnosed by the two methods; (2) The risk of malnutrition exhibited a U-shaped trend with increasing SII, indicating that both low and high SII could increase the risk of malnutrition. To the best of our knowledge, this study represented the first nationally representative investigation of the association between SII and the risk of malnutrition among hospitalized patients in China.

A growing body of evidence indicated a crucial role of inflammation in initiating malnutrition during the disease period. First, the presence of elevated levels of inflammatory markers was associated with a concurrent decrease in food consumption. Pourhassan et al. demonstrated that a CRP level of ≥3.0 mg/dL was associated with a reduction in food intake during last the 3 months in two-thirds of hospitalized geriatric patients and therefore indicative of a high risk of malnutrition (30). This mechanism was related to the acute inhibition of Agouti-Related Peptide (AgRP) which produces neuronal activity by inflammatory cytokines (31). Second, the inflammatory response affected the absorption of nutrients due to the excessive release of inflammatory factors that caused injury to the intestinal mucosa (32–34). The inflammatory microenvironment altered intercellular connections among epithelial cells through direct and indirect mechanisms. This modification compromised the integrity of the epithelial barrier, making the body more susceptible to excessive exposure to microbial antigens. Consequently, this susceptibility triggered the aggregation of leukocytes and the release of soluble mediators, ultimately leading to mucosal damage (35). Third, inflammatory factors had the potential to interfere with metabolic processes, resulting in abnormalities in protein, lipid, and carbohydrate metabolism, subsequently influencing energy balance and nutrient utilization (12, 36, 37). These metabolic irregularities presented symptoms such as weight loss and reduced muscle mass, further exacerbating malnutrition (38, 39).

As a composite indicator, SII complicated the analysis of U-shaped relationships in this study. Firstly, emerging evidence suggests that the release of various inflammatory mediators, such as platelet factor 4 (PF4) and thromboxane A2, by platelets could induce an increase in vascular permeability, facilitate infiltration of inflammatory mediators, and trigger inflammatory reactions (40, 41). Furthermore, platelet-released soluble CD (cluster of differentiation)-84 could improve the motility of wild-type CD4+ T cells *in vitro*, potentially triggering an inflammatory response (42). Secondly, neutrophils, crucial components of the immune system, undergo an increase in numbers during pathological conditions. This increase led to increased immune system activation and the subsequent release of a significant amount of inflammatory mediators that induce inflammation. In both the zebrafish tail injury and the murine acute lung injury models of neutrophilic inflammation, SEMA3F overexpression resulted in delayed resolution of inflammation, characterized by slower neutrophil migration speeds and increased retention of neutrophils within tissues (43). Similarly, lymphodepletion might affect the immune system’s ability to mount a response to infection, thereby increasing the body’s susceptibility to infections and inflammation (44). The presence of the three indicators of functional disparity and diversity was likely to be the underlying cause of the U-shaped relationship. It was not the first time that SII had been reported to demonstrate a U-shaped correlation with the risk of other diseases (45, 46). The U-shaped relationship provided a more precise reflection of the impact of immune inflammation levels on disease progression.

Recent studies have highlighted a significant correlation between SII and malnutrition, revealing that higher SII is often associated with worsened nutritional status and increased risk of malnutrition-related complications. For instance, research on patients with ischemic stroke demonstrated a negative correlation between SII and various nutritional indices such as the prognostic nutritional index and the CONUT score. Higher SII correlated with poorer nutritional statuses, suggesting that SII could serve as a useful marker for assessing malnutrition in these patients (47). Another study focusing on elderly patients with hip fractures found that higher SII values were significantly associated with increased 1-year mortality rates. This study also noted that patients with higher SII levels had lower GNRI values, further reinforcing the link between high systemic inflammation and poor nutritional outcomes (20). Moreover, research on diabetic patients indicated that elevated SII was associated with a higher incidence of diabetic retinopathy, illustrating that systemic inflammation could complicate chronic conditions and potentially worsen nutritional status due to prolonged disease burden (48). These findings collectively underscore the potential of SII as a predictive marker for malnutrition and its associated risks, offering a valuable tool for early intervention and better management of patient outcomes in clinical settings.

Finally, in discussing the discrepancy between the NRS 2002 + GLIM criteria and the CONUT score in assessing malnutrition severity, several factors need to be considered. Firstly, the NRS 2002 + GLIM criteria rely on clinical judgment and evaluate multiple dimensions including weight change, food intake, disease severity, body composition, and inflammatory markers (49), whereas the CONUT score primarily assesses serum albumin, total cholesterol, and lymphocyte count to gauge nutritional status and immune function (27). This divergence in assessment indicators likely contributes to inconsistent findings between the two methods. Secondly, the temporal aspects differ between these tools. The NRS 2002 + GLIM criteria focus on short-term nutritional changes and acute phase responses (50), whereas the CONUT score reflects chronic nutritional status over time (51). Such variation in assessment duration may lead to discrepancies in identifying malnutrition severity. Moreover, the heterogeneity among patient groups, including different disease types, durations, and treatments, influences the outcomes of malnutrition assessments. For instance, patients with chronic diseases may be classified as severely malnourished according to the NRS 2002 + GLIM criteria but not by the CONUT score. While the GLIM criteria exhibit high sensitivity, the CONUT score demonstrates effectiveness in predicting clinical outcomes, suggesting potential complementarity in acute care settings. In conclusion, further research should validate and optimize these assessment tools to enhance consistency and accuracy in clinical applications.

The strengths of this study were notable in several aspects. First, the study had a large sample size, which provided convincing data support and enhanced the reliability and representativeness of the results. Second, it encompassed a wide range of variables, including demographics, disease diagnosis, physical and laboratory examination data, thus offering a comprehensive understanding of the multifaceted characteristics of the study subjects. In terms of study design, a multistage stratified cluster random sampling method was employed to mitigate sample selection bias and improve internal validity. Third, two diagnostic methods were utilized for the assessment of malnutrition to ensure the accuracy of the result through multiple validations. Additionally, comprehensive adjustments for possible confounders were made to eliminate external interference in research conclusions and enhance both internal and external validity.

Several limitations should also be acknowledged. Due to the cross-sectional nature of this study, we were unable to establish a causal relationship between SII and malnutrition; instead, we were able to provide only information on their correlation. To gain a more comprehensive understanding of the dynamic relationship between them, future research might consider longitudinal studies or clinical trials. On the other hand, there were instances of missing values in the original database that impacted our ability to diagnose malnutrition accurately and potentially resulted in an underestimated prevalence. To minimize the occurrence of missing data, it was recommended to employ additional methods for data collection in future studies.

## 5 Conclusion

In conclusion, our study has revealed a statistically significant U-shaped correlation between SII and the risk of malnutrition in hospitalized Chinese patients. This finding provides valuable insights into the relationship between SII and malnutrition prognosis. The observed U-shaped correlation exhibits the complexity of this association. To substantiate and further understand this relationship, more high-quality prospective studies with larger sample sizes and extended follow-up periods are warranted. In addition, exploring potential mechanisms will contribute to a more comprehensive understanding of the impact of SII on the outcomes of malnutrition.

## Data Availability

The original contributions presented in the study are included in the article/Supplementary material, further inquiries can be directed to the corresponding authors.
