# Corrigendum: Association of systemic immune-inflammation index with malnutrition among Chinese hospitalized patients: a nationwide, multicenter, cross-sectional study

**DOI:** 10.3389/fnut.2024.1495183

**Published:** 2024-10-03

**Authors:** Mengyuan Chen, Shu-an Wang, Jiayao Yang, Jiawang Bai, Jingyue Gu, Haolong Luo, Xudong Zhang, Yan Han, Jihong Shao, Yan Xu, Shuyan Guo, Xiangmei Ren

**Affiliations:** ^1^Department of Nutrition, School of Public Health, Xuzhou Medical University, Xuzhou, Jiangsu, China; ^2^Jiangsu Engineering Research Center of Biological Data Mining and Healthcare Transformation, Xuzhou Medical University, Xuzhou, Jiangsu, China; ^3^Department of Clinic Nutrition, Nanjing Drum Tower Hospital, The Affiliated Drum Tower Hospital Clinical College of Xuzhou Medical University, Nanjing, Jiangsu, China; ^4^Department of Clinic Nutrition, The Affiliated Hospital of Xuzhou Medical University, Xuzhou, China; ^5^National Institute of Hospital Administration, National Health Commission, Beijing, China; ^6^Jiangsu Provincial Center for Disease Control and Prevention, Nanjing, Jiangsu, China

**Keywords:** systemic immune-inflammation index, malnutrition, NRS 2002, GLIM, CONUT score

In the published article, there was an error in the affiliation numbers assigned to the authors. The authors, Xudong Zhang and Shuyan Guo, were assigned affiliation 1 and 5. Their correct affiliation is affiliation 5 only.

The authors apologize for this error and state that this does not change the scientific conclusions of the article in any way. The original article has been updated.

