# Supplementary material for: Association of systemic immune-inflammation index with malnutrition among Chinese hospitalized patients: a nationwide, multicenter, cross-sectional study
Source: Front Nutr. 2024 Aug 27;11:1375053. doi: 10.3389/fnut.2024.1375053 (PMC11383780; doi:10.3389/fnut.2024.1375053)
Supplement: Supplementary file 1 [file Data_Sheet_1.docx]

**Association of Systemic Immune-Inflammation Index with Malnutrition in Chinese Hospitalized Patients:**

**A Nationwide, Multicenter, Cross-Sectional Study**

**Supplementary Materials**

**Table S1**．Nutrition Risk Screening 2002. …………………………………………..……2

**Table S2．**The basic scoring of NRS 2002 in hospitalized patients. ….……………………3

**Table S3．**Global Leadership Initiative on Malnutrition (GLIM) Criteria. …. ……………4

**Table S4**．Measurement methods of physical examination indicators. …………………. 5

**Table S5**．Normal reference value range of some indexes in our study. ………………... 6

**Figure S1**．ROC curves for seven different categories of diseases. ……………..…………7

**Table S6**．Baseline characteristics of the study population stratified by the CONTU score.................................................................................................9

**Table S7．**Multiple model to adjust the odds ratio of different severity malnutrition. ..... 11

**Table S1**．Nutrition Risk Screening 2002.

| **socre** | **Nutrition Risk Screening（NRS 2002）grading rules** | | |
| --- | --- | --- | --- |
|  | Nutritional impairment status score | Disease severity score | Age score |
| 0 | Normal nutritional status. | Normal nutrient requirements. | <70 years old. |
| 1 | Weight loss >5% within 3 months, or food intake below 25 to 50% of normal requirements during the precedingweek. | Presence of general malignant tumors, hip fractures, long-term hemodialysis, diabetes mellitus, chronic diseases with acute complications (liver cirrhosis, COPD). | ≥70 years old. |
| 2 | Weight loss >5% within 2 months, or food intake below 50 to 75% of normal requirements during the preceding week. | Major abdominal surgery, stroke, severe pneumonia, or hematological malignancies. |  |
| 3 | Weight loss >5% within 1 month (>5% within 3 months) or BMI <18.5 + inpaired genegal condition or food intake below 50 to 75% of normal requirements during the preceding week. | Head injury, bone marrow transplantation, intensive care patients (APACHEⅡ >10) . |  |
| Nutritional impairment status score□ + Disease severity score□ + Age score□ = Total score□ | | | |

**Table S2．**The basic scoring of NRS 2002 in hospitalized patients

| Score(s) | Frequency, *n* | Percentage, *%* | Effective percentage, *%* | Cumulative percentage, *%* |
| --- | --- | --- | --- | --- |
| 0 | 6039 | 15.0 | 15.0 | 15.0 |
| 1 | 16571 | 41.0 | 41.0 | 56.0 |
| 2 | 9139 | 22.6 | 22.6 | 78.6 |
| 3 | 4446 | 11.0 | 11.0 | 89.6 |
| 4 | 2669 | 6.6 | 6.6 | 96.2 |
| 5 | 1151 | 2.9 | 2.9 | 99.1 |
| 6 | 353 | 0.9 | 0.9 | 100.0 |
| 7 | 11 | 0.0 | 0.0 | 100.0 |
| Total | 40379 | 100.0 | 100.0 | - |

**Table S3**．Global Leadership Initiative on Malnutrition (GLIM) Criteria.

| **Phenotypic criteria** | | | | | |
| --- | --- | --- | --- | --- | --- |
| Weight Loss (%) | Low BMI (kg/m^2^) | | | Muscle Mass Reduction (kg/m^2^) | |
| >5% within the past 6 months, or >10% beyond 6 months. | Asia: <18.5 if <70 years, or <20.0 if ≥70 years. | | | Male: < 7.0;  Female: < 5.7. | |
| **Etiologic Criteria** | | | | | |
| Food Intake or Assimilation Reduction | | Inflammation | | | |
| ≤50% of energy requirements >1 week, or any reduction for >2 weeks, or any chronic gastrointestinal condition that adversely impacts food assimilation or absorption. | | 1) The presence of chronic inflammation-related disease burden, such as heart failure, chronic obstructive pulmonary disease, chronic kidney disease, chronic liver disease, and malignancies;  2) Clinical indicators of acute or severe inflammatory states, such as fever (>38°C), elevated C-reactive protein (CRP) levels (>3 mg/L), and hypoalbuminemia (<30 g/L). | | | |
| **Severity Grading** | | | | | |
|  | Weight Loss (%) | | Low BMI (kg/m^2^) | | Muscle Mass Reduction (kg/m^2^) |
| Moderate Malnutrition ^a^ | 5-10% within the past 6 months or >10-20% beyond 6 months. | | <20.0 if <70 years, or <22.0 if ≥70 years. | | Mild to moderate reduction ^b^. |
| Severe Malnutrition ^a^ | >10% within the past 6 months or >20% beyond 6 months. | | <18.5 if <70 years, or <20.0 if ≥70 years. | | Severe reduction ^b^. |

^a^ Requires 1 phenotypic criterion that meets this grade;

^b^ Based on validated measurement techniques.

**Table S4**．Measurement methods of physical examination indicators.

| **Physical Examination** | **Specific Calculation and Measurement Methods** |
| --- | --- |
| BMI  (kg/m^2^) | BMI was calculated by dividing the weight in kilograms by the square of the height in meters. The reference standards were as follows: Underweight (BMI<18.5kg/m^2^), Normal (18.5≤BMI＜24.0kg/m^2^), Overweight (24.0≤BMI<28.0kg/m^2^), and Obese (BMI≥28kg/m^2^). |
| Waist circumference  (cm) | The midpoint between the upper edge of the hip bone and the lower edge of the ribs on both sides of the subject was measured during a calm exhalation, and the reading was accurate to 0.1 cm. |
| Hip circumference  (cm) | The hip circumference refers to the horizontal measurement around the most prominent part of the buttocks. |
| Biceps circumference  (cm) | The arms should naturally hang down, with the brachial biceps positioned at the thickest part of the upper arm. The subject's palms should rest on the sides of their thighs. Accurate measurement to 0.1cm is required for determining the circumference at the midpoint of each upper arm. The normal range of upper arm circumference was ≥24.75cm for men and ≥23.22cm for women. |
| Calf circumference  (cm) | A flexible tape measure was positioned horizontally around the thickest part of the subject's calf with a precision of 0.1cm, and the normal range of calf circumference was ≥29cm. |
| Grip strength  (kg) | The side of the hand should be checked, and spring grips should be used. The grip strength should be measured while standing and stretching the cubits. The maximum power should be measured with the opposite hand at least 2 times, and the largest reading should be selected. The normal range of grip strength was ≥29.6kg in men and ≥18.6kg in women. |

**Table S5**．Normal reference value range of some indexes in our study.

| **Index** | **Normal Reference Value Range** |
| --- | --- |
| WBC, *×10^9^/L* | 4.0-11.0 |
| NEUT *%* | 50-70 |
| LY *%* | 20-40 |
| RBC, *×10^12^/L* | Male: ≥4.5 |
|  | Female: ≥4.0 |
| Hb, *g/L* | Male: ≥120 |
|  | Female: ≥110 |
| BPC, *×10^9^/L* | 100-300 |
| TC, *mmol/L* | <4.65 |
| TG, *mmol/L* | 0.45-1.70 |
| ALB, *g/L* | ≥35 |

|  | Without Malnutrition | With Malnutrition |
| --- | --- | --- |
| Tumor | 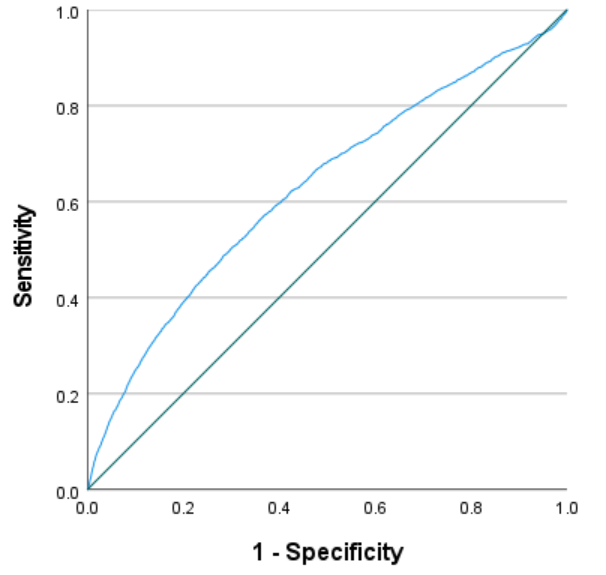 | 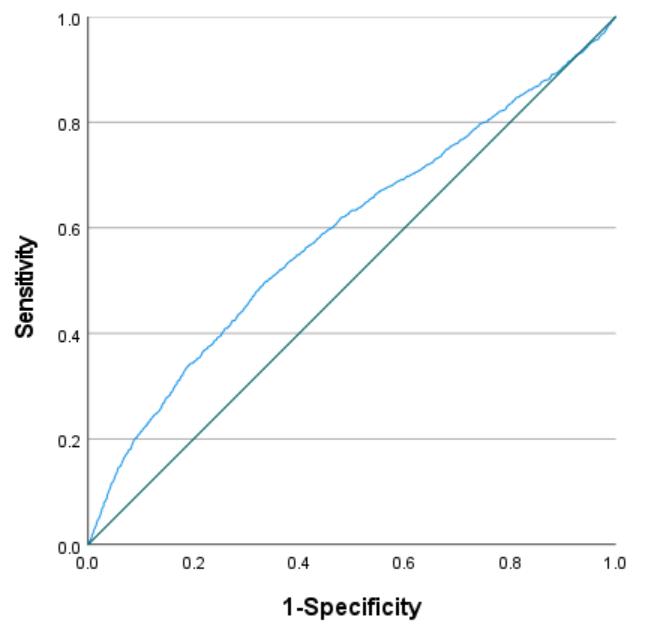 |
|  | AUC: 0.628 | AUC: 0.590 |
| Endocrine  disease | 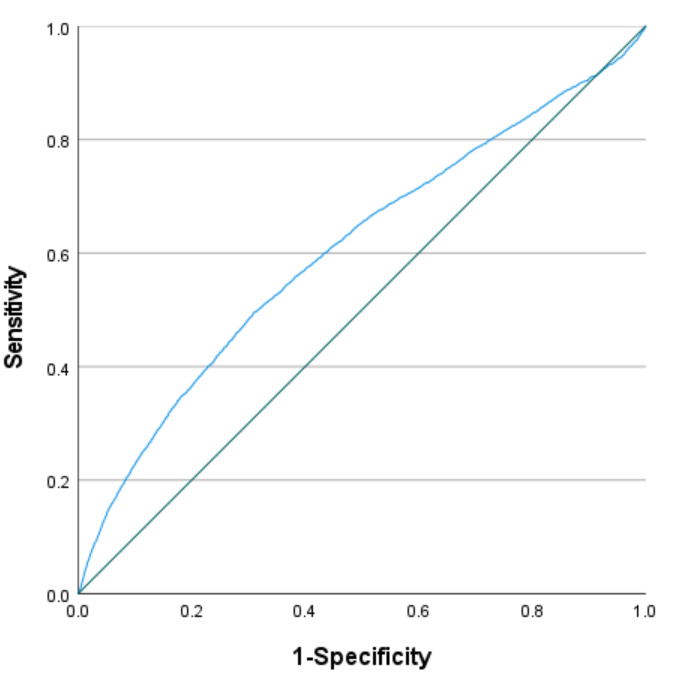 | 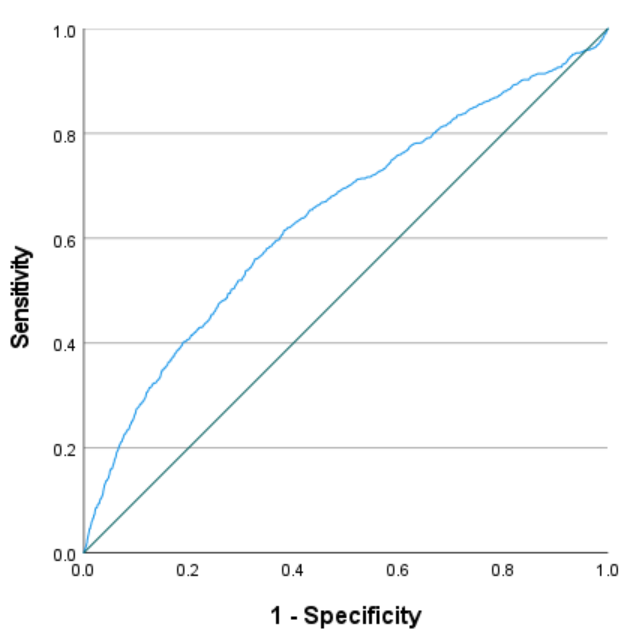 |
|  | AUC: 0.607 | AUC: 0.642 |
| Nervous  disease | 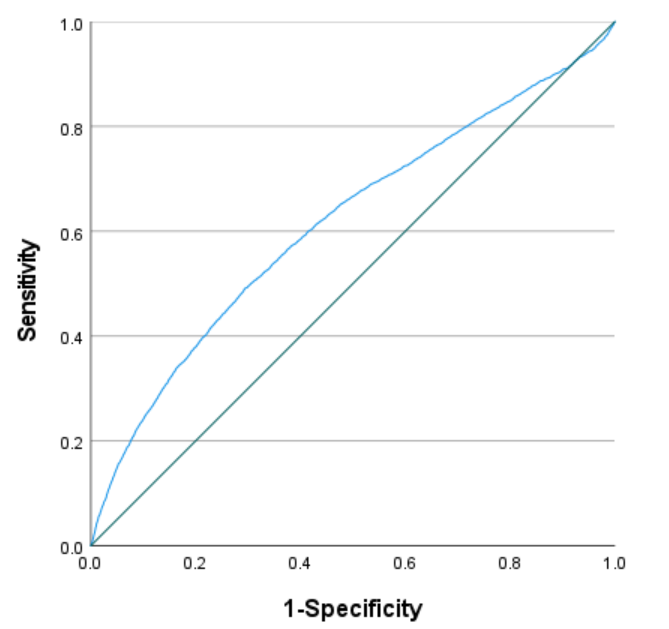 | 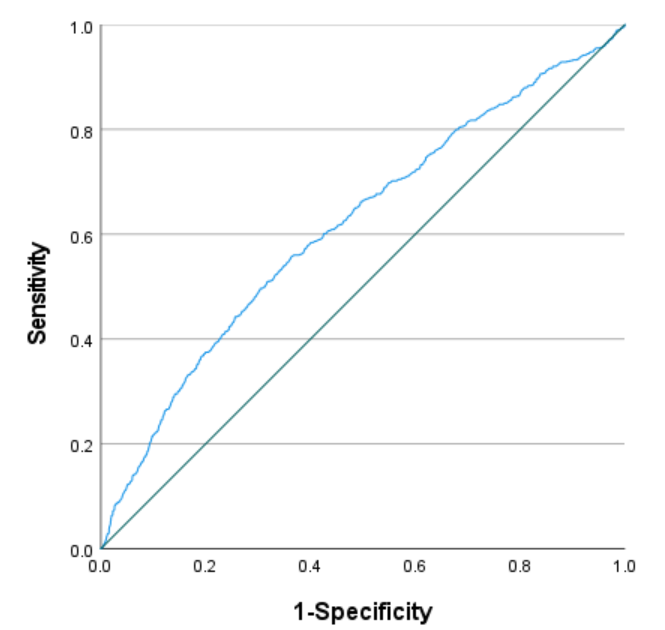 |
|  | AUC: 0.616 | AUC: 0.616 |
| Circulation  disease | 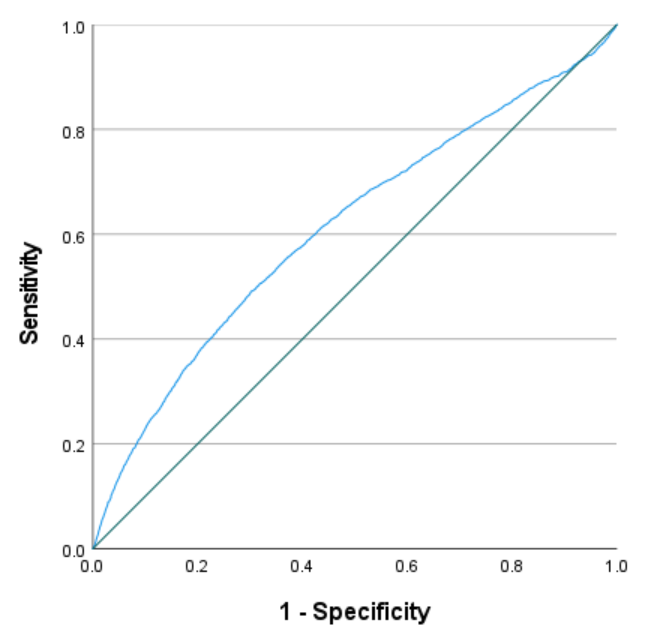 | 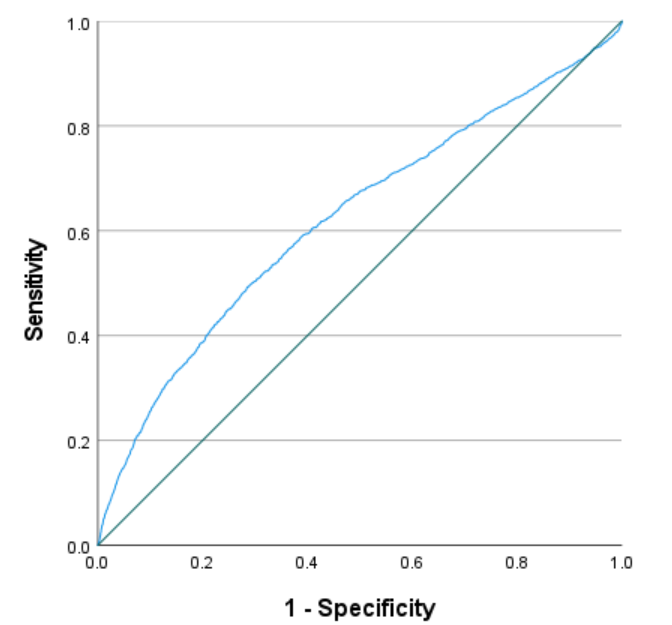 |
|  | AUC: 0.612 | AUC: 0.613 |
| Respiratory  disease | 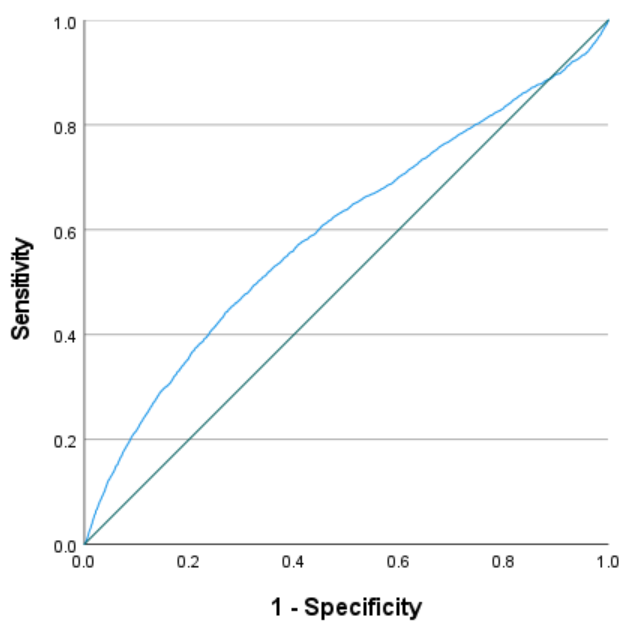 | 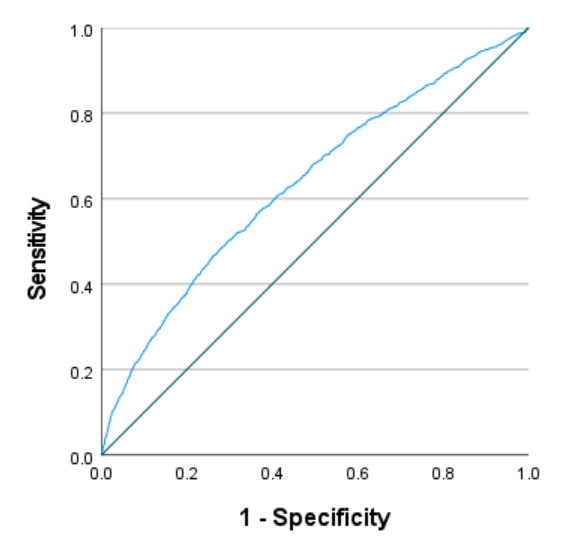 |
|  | AUC: 0.596 | AUC: 0.635 |
| Digestive  disease | 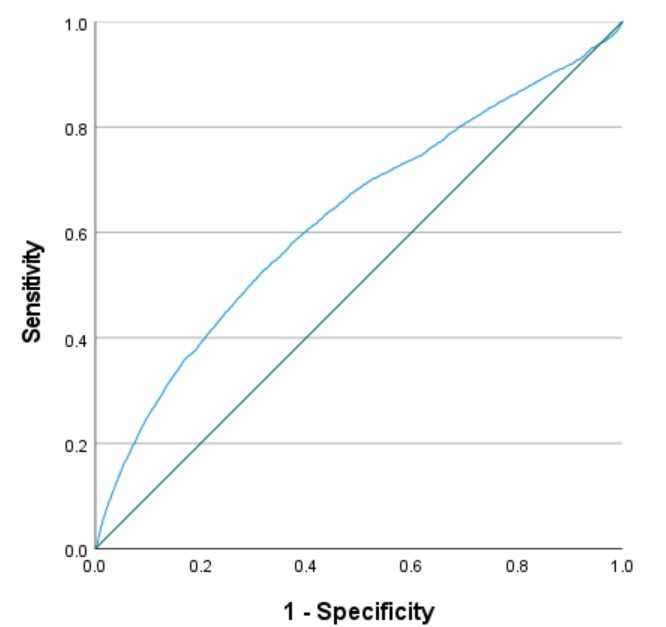 | 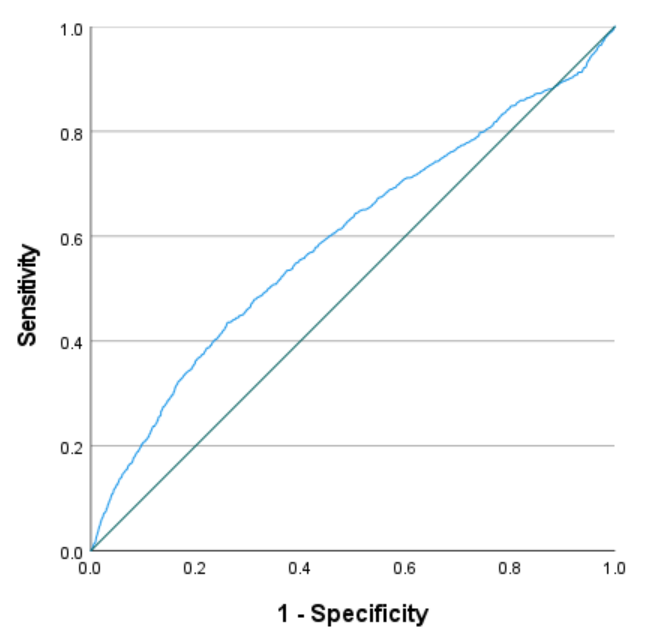 |
|  | AUC: 0.628 | AUC: 0.594 |
| Genitourinary  disease | 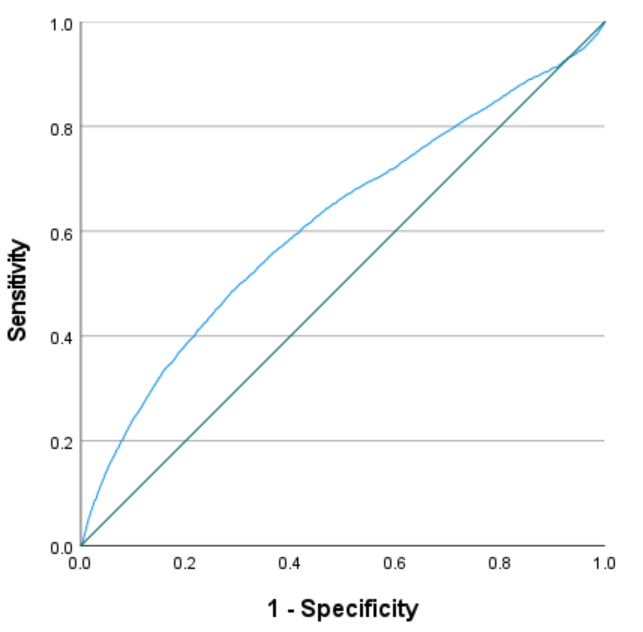 | 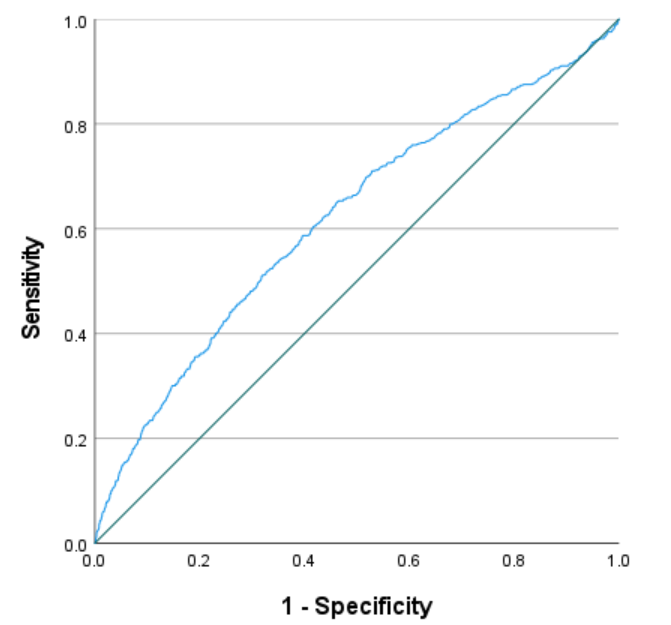 |
|  | AUC: 0.616 | AUC: 0.619 |

**Figure S1**．ROC curves for seven different categories of diseases

**Table S7．**Baseline characteristics of the study population stratified by the CONTU score.

| **Characteristics** | **Undernutrition Degree** | | | |
| --- | --- | --- | --- | --- |
|  | **None** | **Mild** | **Moderate** | **Severe** |
| **Participants, *n(%)*** | 16202 (40.1) | 18162 (45.0) | 5286 (13.1) | 729 (1.8) |
| **lnSII** | 6.07±0.59 | 6.41±0.81 | 6.77±1.03 | 6.99±1.22 |
| **SII, *n(%)*** |  |  |  |  |
| Q1(<332) | 5236 (32.3) | 3872 (21.3) | 864 (16.3) | 113 (15.5) |
| Q2(332-514) | 5133 (31.7) | 4243 (23.4) | 673 (12.7) | 76 (10.4) |
| Q3(515-874) | 4056 (25.0) | 4774 (26.3) | 1138 (21.5) | 111 (15.3) |
| Q4(>875) | 1777 (11.0) | 5273 (29.0) | 2611 (49.5) | 429 (58.8) |
| **Gender, *n(%)*** |  |  |  |  |
| Male | 8328 (51.4) | 10633 (58.5) | 3439 (65.1) | 494 (67.8) |
| Female | 7874 (48.6) | 7529 (41.5) | 1847 (34.9) | 235 (32.2) |
| **Age, *years*** | 56.8±14.0 | 60.5±14.3 | 64.1±14.5 | 65.1±14.3 |
| **Education Level, *n(%)*** |  |  |  |  |
| Never | 1532 (9.5) | 1944 (10.7) | 705 (13.3) | 122 (16.7) |
| Primary or junior high school | 7617 (47.0) | 9561 (52.7) | 2942 (55.7) | 403 (55.3) |
| High school or vocational school | 4237 (26.1) | 4389 (24.2) | 1161 (22.0) | 143 (19.6) |
| Bachelor degree or above | 2816 (17.4) | 2268 (12.5) | 478 (9.0) | 61 (8.4) |
| **Physical Examination** |  |  |  |  |
| BMI, *kg/m^2^* | 24.7±3.9 | 23.6±3.8 | 22.5±4.0 | 21.5±4.0 |
| Waist circumference, *cm* | 88.0 (81.0, 95.5) | 86.0 (79.0, 93.7) | 84.0 (76.2, 92.0) | 82.0 (74.4, 90.0) |
| Hip circumference, *cm* | 96.0 (91.0, 101.2) | 94.2 (89.0, 100.0) | 91.9 (86.0, 98.0) | 90.0 (84.0,95.0) |
| Biceps circumference, *cm* | 28.5±3.7 | 27.6±3.8 | 26.2±4.0 | 25.0±4.3 |
| Calf circumference, *cm* | 34.6±4.3 | 33.7±4.4 | 32.2±4.9 | 31.2±5.6 |
| Grip, *kg* | 26.6 (19.7, 34.9) | 25.3 (18.6, 32.8) | 22.7 (16.0, 30.0) | 20.6 (14.5, 28.3) |
| **Laboratory Examination** |  |  |  |  |
| WBC, *×10^9^/L* | 6.43 (5.37, 7.80) | 5.83 (4.60, 7.55) | 5.95 (4.30, 8.40) | 6.12 (4.16, 9.40) |
| RBC, *×10^12^/L* | 4.53 (4.16, 4.91) | 4.25 (3.81, 4.67) | 3.69 (3.15, 4.20) | 3.12 (2.58, 3.67) |
| Hb, *g/L* | 137 (126, 150) | 129 (115, 142) | 111 (93, 127) | 94 (75, 112) |
| TC, *mmol/L* | 4.96 (4.38, 5.60) | 3.97 (3.35, 4.63) | 3.42 (2.85, 4.12) | 2.79 (2.32, 3.36) |
| TG, *mmol/L* | 1.50 (1.06, 2.18) | 1.17 (0.85, 1.68) | 1.06 (0.77, 1.53) | 0.94 (0.69, 1.39) |
| ALB, *g/L* | 41.6 (38.9, 44.4) | 39.6 (36.6, 42.7) | 32.5 (29.3, 34.8) | 24.8 (22.4, 27.7) |
| **Tumor, *n(%)*** |  |  |  |  |
| No | 13382 (82.6) | 13765 (75.8) | 3835 (72.6) | 547 (75.0) |
| Yes | 2820 (17.4) | 4397 (24.2) | 1451 (27.4) | 182 (25.0) |
| **Endocrine disease, *n(%)*** |  |  |  |  |
| No | 11322 (69.9) | 13814 (76.1) | 4234 (80.1) | 604 (82.9) |
| Yes | 4880 (30.1) | 4348 (23.9) | 1052 (19.9) | 125 (17.1) |
| **Nervous disease, *n(%)*** |  |  |  |  |
| No | 13273 (81.9) | 15013 (82.7) | 4664 (88.2) | 659 (90.4) |
| Yes | 2929 (18.1) | 3149 (17.3) | 622 (11.8) | 70 (9.6) |
| **Circulation disease, *n(%)*** |  |  |  |  |
| No | 10377 (64.0) | 11345 (62.5) | 3531 (66.8) | 549 (75.3) |
| Yes | 5825 (36.0) | 6817 (37.5) | 1755 (33.2) | 180 (24.7) |
| **Respiratory disease, *n(%)*** |  |  |  |  |
| No | 14029 (86.6) | 14849 (81.8) | 3918 (74.1) | 523 (71.7) |
| Yes | 2173 (13.4) | 3313 (18.2) | 1368 (25.9) | 206 (28.3) |
| **Digestive disease, *n(%)*** |  |  |  |  |
| No | 12882 (79.5) | 14615 (80.5) | 3940 (74.5) | 444 (60.9) |
| Yes | 3320 (20.5) | 3547 (19.5) | 1346 (25.5) | 285 (39.1) |
| **Genitourinary disease, *n(%)*** |  |  |  |  |
| No | 13900 (85.8) | 15535 (85.5) | 4212 (79.7) | 607 (83.3) |
| Yes | 2302 (14.2) | 2627 (14.5) | 1074 (20.3) | 122 (16.7) |

Data were n (%) or mean ± SD; SII, systemic immune-inflammatory index; BMI, body mass index; WBC, white blood cell count; RBC, red blood cell count; Hb, hemoglobin; TC, total cholesterol; TG, triglyceride; ALB, albumin.

**Table S8．**Multiple model to adjust the odds ratio of different severity malnutrition.

| Models | OR (95%CI) | | |
| --- | --- | --- | --- |
|  | Mild malnutrition | Moderate malnutrition | Severe malnutrition |
| Crude model (Ref, Q_1_<332) | |  |  |
| Q_2_ (332-514) | 1.12 (1.01, 1.19) | 0.80 (0.71, 0.89) | 0.69 (0.51, 0.92) |
| Q_3_ (515-874) | 1.59 (1.50, 1.69) | 1.70 (1.54, 1.87) | 1.27 (0.97, 1.65) |
| Q_4_ (>875) | 4.01 (3.75, 4.30) | 8.90 (8.11, 9.78) | 11.19 (9.03, 13.86) |
| Model Ⅰ (Ref, Q_1_<332) | |  |  |
| Q_2_ (332-514) | 1.12 (1.04, 1.17) | 0.78 (0.70, 0.87) | 0.67 (0.50, 0.90) |
| Q_3_ (515-874) | 1.58 (1.49, 1.68) | 1.67 (1.51, 1.84) | 1.24 (0.95, 1.61) |
| Q_4_ (>875) | 4.00 (3.73, 4.28) | 8.71 (7.92, 9.85) | 10.86 (8.76, 13.47) |
| Model Ⅱ (Ref, Q_1_<332) | |  |  |
| Q_2_ (332-514) | 1.12 (1.06, 1.19) | 0.79 (0.71, 0.88) | 0.68 (0.51, 0.91) |
| Q_3_ (515-874) | 1.58 (1.49, 1.68) | 1.61 (1.46, 1.78) | 1.18 (0.90, 1.54) |
| Q_4_ (>875) | 3.87 (3.61, 4.14) | 7.73 (7.02, 8.52) | 9.00 (7.24, 11.20) |
| Model Ⅲ (Ref, Q_1_<332) | |  |  |
| Q_2_ (332-514) | 1.13 (1.07, 1.20) | 0.80 (0.71, 0.89) | 0.71 (0.53, 0.96) |
| Q_3_ (515-874) | 1.57 (1.48, 1.67) | 1.62 (1.46, 1.79) | 1.24 (0.95, 1.62) |
| Q_4_ (>875) | 3.80 (3.54, 4.07) | 7.63 (6.91, 8.42) | 9.48 (7.60, 11.84) |

Model I: Adjusted for sociodemographic characteristics.

Model II: Adjusted for Model I and physical examination characteristics.

Model III: Adjusted for Model II and diease characteristics.
